# Chromosomal fusions, but not chromosomal inversions, activate a PCH-2 dependent checkpoint that promotes crossover formation in *C. elegans*

**DOI:** 10.17912/micropub.biology.000839

**Published:** 2023-06-19

**Authors:** Bhumil Patel, Maryke Grobler, Needhi Bhalla

**Affiliations:** 1 Molecular, Cellular, and Developmental Biology, University of California, Santa Cruz, Santa Cruz, California, United States

## Abstract

Meiotic crossovers promote accurate chromosome segregation during gametogenesis. In *C. elegans* , a highly conserved AAA ATPase, PCH-2, ensures that homologous chromosomes have at least one crossover, preventing meiotic defects. PCH-2 localizes to meiotic chromosomes and this localization is extended when there are defects in meiotic recombination, suggesting a role in responding to defects. Here, we show that, unlike in other systems, PCH-2 does not persist on meiotic chromosomes when there are chromosomal inversions but does persist when there are whole chromosome fusions. Moreover, this persistence correlates with an increase in crossovers, demonstrating that PCH-2’s localization to chromosomes promotes crossover formation.

**Figure 1.**
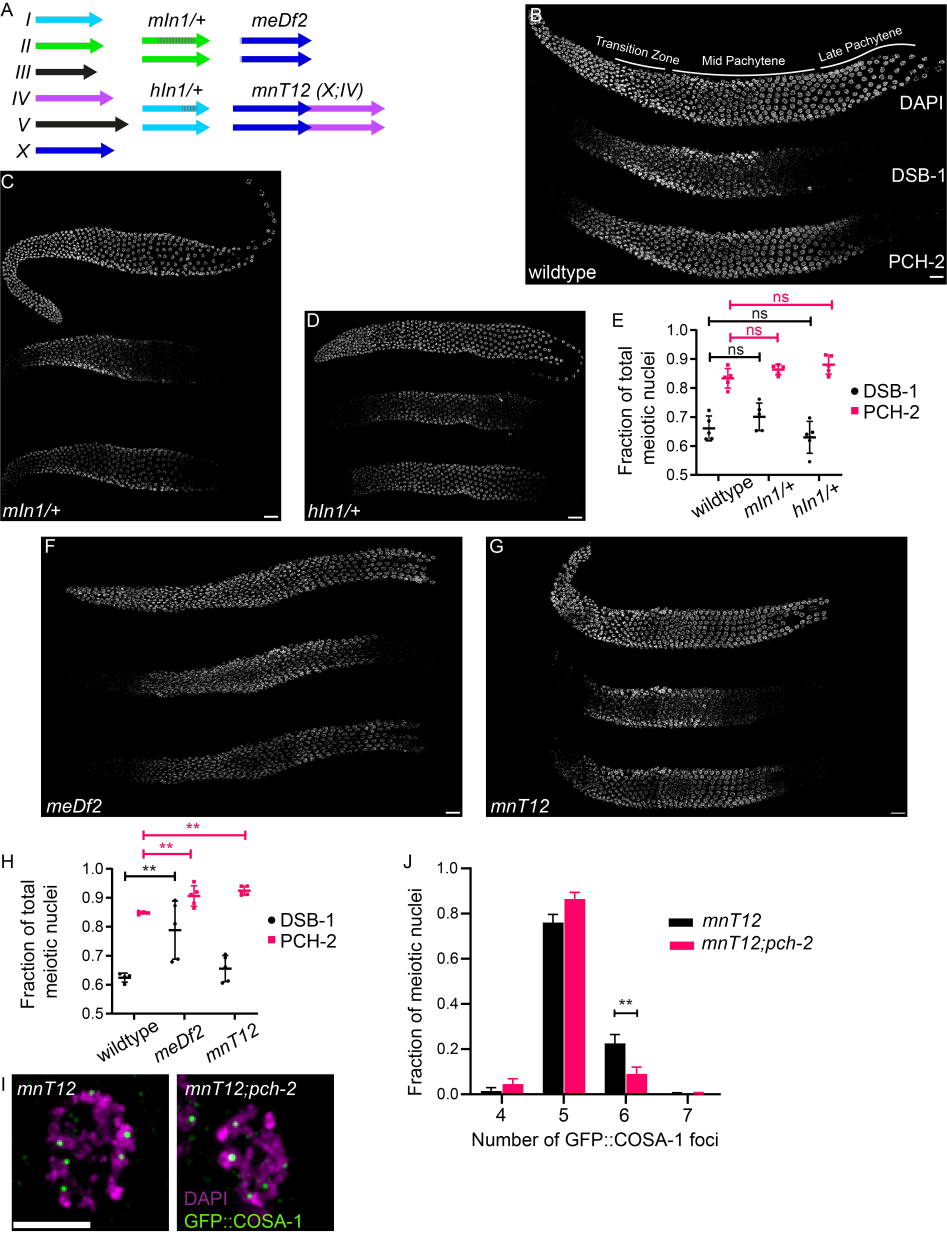
Figure 1. A. Schematic of six chromosomes in wildtype worms drawn to scale, as well as in backgrounds with chromosomal rearrangements, *mIn1/+* , *hIn1/+* , *meDf2* and *mnT12* . Arrows indicate the right end of the chromosomes and dashed gray lines indicate portions of chromosomes inverted in *mIn1/+* and *hIn1/+* . *meDf2* is a terminal or near-terminal deletion of the left portion of the X chromosome, with some ambiguity regarding its breakpoint (shaded portion), and *mnT12* is a fusion between chromosomes X and IV. Representative images of wildtype (B), *mIn1/+* (C), and *hIn1/+* (D) hermaphrodite germlines stained with DAPI (top) and antibodies against DSB-1 (middle) and PCH-2 (bottom). E. Fraction of total meiotic nuclei in the germline stained with DSB-1 and PCH-2 in wildtype, *mIn1/+* , and *hIn1/+* germlines (n=5 per genotype). No significant difference (ns) is observed between wildtype, *mIn1/+* , and *hIn1/+* . Representative images of *meDf2* (F) and *mnT12* hermaphrodite germlines (G) stained with DAPI (top) and antibodies against DSB-1 (middle) and PCH-2 (bottom). H. Fraction of total meiotic nuclei in the germline stained with DSB-1 and PCH-2 in wildtype, *meDf2* , and *mnT12* germlines (n=4 for wildtype and n=5 for *meDf2* and *mnT12* ). I. Representative images of meiotic nuclei stained with DAPI (magenta) and GFP::COSA-1 (green). The nucleus in *mnT12* has six GFP::COSA-1 foci while the nucleus in *mnT12;pch-2* has five. Scale bar indicates 2 microns. J. Histogram representing the fraction of meiotic nuclei with four, five, six, and seven GFP::COSA-1 foci in *mnT12* (n=483 nuclei) and *mnT12;pch-2* mutants (n=446 nuclei). In all graphs, error bars indicate standard deviation and ** indicates a p value < 0.001. Unless otherwise stated, scale bars in images indicate 8 microns.

## Description

Meiosis is a specialized form of cell division that generates haploid sex cells, such as sperm and eggs. During this process, meiotic chromosomes undergo a series of complex events that ultimately lead to exchanges between the maternal and paternal copies of each chromosome (homologs), generating new genetic combinations. Proper segregation of homologous chromosomes during the first round of division (meiosis I) also relies on these genetic exchange events, known as a crossovers or chiasmata, which link homologs and promote their bi-orientation on the meiosis I spindle. Defects in meiotic chromosome segregation can result in aneuploid embryos, which account for a substantial proportion of miscarriages and infertility in humans (Nagaoka et al., 2012), and can cause genetic disorders such as Down Syndrome.

Crossovers form when programmed DNA double-strand breaks (DSBs) are repaired using a conserved repair pathway called homologous recombination. During meiosis, homologous recombination is biased towards the homolog to promote the formation of chiasmata (Bhalla & Dernburg, 2008) . In addition, the number and distribution of crossovers are tightly controlled to ensure that each homologous pair has at least one crossover (assurance) and that they are widely spaced (interference) (Gray & Cohen, 2016). In addition to these regulatory pathways, feedback and surveillance mechanisms monitor intermediates and respond to errors in recombination. For example, in both budding yeast and mice, defects in the ability to repair DSBs using the homolog as a repair template produce an increase in DSB formation, presumably to promote crossover formation (Kauppi et al., 2013; Thacker et al., 2014). A similar response has been observed in *C. elegans* (Rosu et al., 2013; Stamper et al., 2013). Because of the spatiotemporal organization of meiotic nuclei in the *C. elegans* germline, we can observe how checkpoints respond to errors and defects in meiosis by studying the localization of factors that regulate recombination during meiotic prophase. In response to a failure to form crossovers, DSB-1 and DSB-2 (DNA Double Strand Break factors 1 and 2) extend their localization on chromosomes (Rosu et al., 2013; Stamper et al., 2013). Since these factors are required for proper numbers of DSBs, this extension promotes more DSBs, and crossover formation, to provide meiotic fidelity in response to recombination defects (Deshong et al., 2014; Rosu et al., 2013; Stamper et al., 2013). In budding yeast and mice, this response is local and chromosome specific (Kauppi et al., 2013; Mu et al., 2020), while in *C. elegans* DSBs increase across the genome, even in nuclei in which a single chromosome pair cannot undertake homologous recombination (Stamper et al., 2013).

In addition to increasing DSBs, another checkpoint response appears to specifically promote crossover formation when there are defects in meiosis. This checkpoint response relies on the evolutionarily ancient AAA-ATPase, PCH-2, which has been shown to respond to defects in recombination in both *C. elegans* and *Drosophila* (Deshong et al., 2014; Joyce & McKim, 2009, 2010). In *Drosophila* , animals heterozygous for chromosomal inversions display the “interchromosomal effect,” in which the barrier these chromosomal rearrangements introduce to recombination produces a PCH2—dependent meiotic delay and a global increase in crossovers across the genome (Joyce & McKim, 2010). A similar response is seen in *C. elegans* , in mutants in which a single pair of chromosomes is unable to undergo homologous recombination (Carlton et al., 2006; Deshong et al., 2014). It’s unclear whether the PCH-2 dependent increase in crossovers observed in *Drosophila* is the result of an increase in DSBs, the promotion of crossover-specific mechanisms downstream of DSB formation, or both. However, in *C. elegans* , experiments suggest that two feedback mechanisms produce the increase in crossovers: one that increases DSBs and is independent of PCH-2, and another that acts downstream of DSB formation to promote crossovers and is dependent on PCH-2 (Deshong et al., 2014). Here, we formally test whether these two feedback responses can be uncoupled and the consequences of activating only the PCH-2 feedback mechanism on crossover formation.

PCH-2 localizes to meiotic chromosomes when chromosomes are competent for meiotic recombination and this localization is dependent on the synaptonemal complex (Deshong et al., 2014). In mutants that are defective in recombination, PCH-2 persists on meiotic chromosomes (Deshong et al., 2014). To determine if PCH-2 responded similarly to chromosomal inversions in *C. elegans* as in *Drosophila* , we tested whether PCH-2 localization persists on meiotic chromosomes in worms that were heterozygous for chromosomal inversions, *mIn1/+* and *hIn1/+* ( Figure 1A ). We also tested whether these inversions activated the DSB-1 dependent feedback mechanism that increases DSBs. *mIn1* is an inversion that spans 8.2 Mb in the middle of Chromosome II and *hIn1* is an inversion that spans 3.3 Mb on the right end of Chromosome I ( Figure 1A ). Both suppress recombination in the region of their rearrangement and show enhanced recombination in the regions outside the rearrangement (Edgley & Riddle, 2001; Zetka & Rose, 1992). It has already been shown that animals heterozygous for *hIn1* do not appear to show any change or increase in crossovers on other chromosomes (Zetka & Rose, 1992), suggesting neither the DSB-1 or PCH-2 dependent checkpoints are active in this background. However, *mIn1* is a substantially larger inversion than *hIn1* ( Figure 1A ), raising the possibility that this inversion may produce a checkpoint response. We stained *mIn1/+* and *hIn1/+* germlines with antibodies against DSB-1 and PCH-2 and quantified the total number of meiotic nuclei that were DSB-1 positive or PCH-2 positive. In wildtype germlines ( Figure 1B ), DSB-1 localization to meiotic chromosomes begins at the entry into meiosis, also called the transition zone, and is removed in mid-pachytene. An average of 66% of meiotic nuclei in wildtype germlines are DSB-1 positive ( Figure 1E ). PCH-2 also localizes to meiotic chromosomes beginning at the transition zone, and is removed later than DSB-1, before late pachytene ( Figure 1B ). An average of 83% of meiotic nuclei in wildtype germlines are PCH-2 positive ( Figure 1E ). In the *mIn1/+* background ( Figure 1C ), an average of 63% meiotic nuclei are DSB-1 positive and an average of 86% are PCH-2 positive ( Figure 1E ). Neither of these averages are significantly different than wildtype. In the *hIn1/+* germlines ( Figure 1D ), localization was similar to *mIn1/+* and wildtype germlines, with an average of 70% of meiotic nuclei that are DSB-1 positive and 88% of meiotic nuclei that are PCH-2 positive ( Figure 1E ). Our results indicate that neither checkpoint is activated in worms that are heterozygous for *mIn1* or *hIn1* inversions. Since this result is different than the one observed in *Drosophila* (Joyce & McKim, 2010), this suggests that different systems may have different thresholds for activation of these meiotic checkpoints.

To determine if more severe changes in karyotypes, like chromosomal fusions, activate either feedback mechanism, we analyzed *mnT12* mutants. In the *mnT12* background, chromosome IV and X are fused end to end, generating a large fusion chromosome that comprises one third of the genome (Sigurdson et al., 1986) ( Figure 1A ). *mnT12* homozygotes produce >99% viable progeny, indicating that segregation errors leading to aneuploidy are rare (Hillers & Villeneuve, 2003). In this analysis, we also included *meDf2* mutants. In *meDf2* mutants, the X chromosome Pairing Center is deleted from X chromosomes, preventing their ability to recombine (MacQueen et al., 2005; Villeneuve, 1994). This defect in recombination activates both the DSB-1 and PCH-2 checkpoints (Deshong et al., 2014; Stamper et al., 2013), producing additional crossovers and disrupting interference, similar to what has been observed in worms with chromosomal fusions (Yokoo et al., 2012). In *meDf2* germlines ( Figure 1F ), an average of 79% of meiotic nuclei are DSB-1 positive, significantly more than the wildtype average of 62% ( Figure 1H ), indicating the activation of the feedback response governed by DSB-1. Similarly, PCH-2 also persists on meiotic chromosomes into late pachytene in *meDf2* germlines: an average of 91% of meiotic nuclei are PCH-2 positive in this mutant background, compared to the average of 85% in wildtype germlines ( Figure 1H ). In contrast, in the *mnT12* background ( Figure 1G ), an average of 66% of meiotic nuclei are DSB-1 positive and 92% of meiotic nuclei are PCH-2 positive ( Figure 1H ). Thus, worms that carry the *mnT12* fusion chromosome activate the PCH-2 dependent meiotic checkpoint but not the DSB-1 dependent checkpoint, demonstrating that these two checkpoint responses can be functionally uncoupled.

In order to test what effect this extension of PCH-2 would have on crossover formation, we generated strains that incorporated a cytological marker for crossovers – COSA1 – fused to GFP (Yokoo et al., 2012). We generated *mnT12;GFP::COSA-1* and *mnT12;pch-2;GFP::COSA-1* strains and quantified the number of GFP::COSA-1 foci per nucleus in each background. Worms carrying *mnT12* primarily exhibited five foci per nucleus, illustrating the potency of crossover interference on this fusion chromosome. However, 22% of meiotic nuclei exhibited six GFP::COSA-1 foci ( Figure 1I ), consistent with the loss of interference reported for this strain (Yokoo et al., 2012). In contrast, nuclei in *mnT12;pch-2* showed a significant decrease in the number of nuclei that harbor six GFP::COSA foci (8%), confirming that activation of this PCH-2 dependent feedback mechanism promotes crossover formation ( Figure 1I and Figure 1J).

Meiotic control of crossovers is highly regulated, and our results provide insight into one layer of this regulation. Here we show that in *C. elegans* , dramatic changes in karyotype, such as whole chromosome fusions, but not inversions, activate a PCH-2 dependent checkpoint to promote crossover formation and disrupt crossover interference in *C. elegans* . Given that PCH-2 acts with the chromosomal axis component and meiotic HORMAD, HIM-3, to promote crossover assurance (Russo et al., 2023), we hypothesize that PCH-2’s persistence on chromosomes allows this ATPase to continue to act on HIM-3, introducing additional crossovers and affecting crossover interference. This hypothesis is strongly supported by previous reports that another hypomorphic allele of *him-3* also disrupts interference (Nabeshima et al., 2004). Thus, PCH-2's regulation of meiotic axis components may contribute to both crossover assurance and interference.

Why does *mnT12* , and not chromosomal inversions, specifically activate this PCH-2 dependent checkpoint to produce extra crossovers? Since these worms do not exhibit defects in recombination other than the loss of interference, we propose that this extensive change in karyotype, where one chromosome is substantially longer than the others, affects the propagation of whatever molecular signal(s) controls crossover assurance and/or interference on chromosomes, activating this checkpoint. A similar response has been reported in Arabidopsis when polyploidy is chemically induced and cytological defects in interference are observed (Morgan et al. 2021), suggesting that these neopolyploids may also activate a PCH-2-dependent checkpoint. Strikingly, evolved, stable polyploids in Arabidopsis restore robust interference (Morgan et al. 2021) and show evidence of adaptive evolution in meiotic HORMADs, which are presumed substrates of PCH-2, and synaptonemal complex components, which recruit PCH-2 to chromosomes (Yant et al. 2013; Wright et al. 2015; Hollister et al. 2012). Since PCH-2 remodels a variety of HORMADs in most systems (Gu et al. 2022), we have proposed that its own evolutionary trajectory might be constrained (Bhalla, 2023) . Further studies focused on identifying the molecular mechanisms through which meiotic HORMADs interact with PCH-2, especially on synapsed or synapsing chromosomes, will help us understand how the regulation of specific HORMADs ensure crossover control during meiosis.

## Methods

***C. elegans*** **strains and genetics:**

The Bristol N2 C. *elegans* strain (Brenner et al. 1974) was used as the wild-type control for all experiments. Strains were maintained on Nematode Growth Media seeded with OP50 bacteria and grown at 20℃ under standard conditions for all immunostaining experiments. Mutations and rearrangements used were as follows:

LG I: *mnDp66, hIn1 [unc-54(h1040)]*

LG II: *pch-2(tm1458), meIs8 [Ppie-1::GFP::cosa-1 + unc-119(+)], mIn1 [unc-4(e120)], let-552(e2542), rol-1(e91)*

LG IV: *mnT12 (X;IV)*

LG X: *meDf2* , *mnT12 (X;IV)*

Specific strains used were KR2151 ( *hIn1[unc-54(h1040)] I),* DR2075 ( *mIn1[unc-4(e120)]/let-552(e2542) rol-1(e91) II),* SP646 ( *mnT12 X;IV* ) , and AV38 ( *mnDp66 X;I;meDf2 X* ) (CGC strain designations). *meDf2* is a terminal deficiency of the left end of the X chromosome that removes the X chromosome pairing center (PC) as well as numerous essential genes. For this reason, homozygous *meDf2* animals also carry a duplication ( *mnDp66* ) that includes these essential genes but does not interfere with normal X chromosome segregation (Herman and Kari, 1989).

**DAPI staining and Immunofluorescence:**

Adult hermaphrodites were fixed and stained 24-26 hours post L4 larval stage as in (Bhalla, 2005) .

For analyzing PCH-2 localization, the following primary antibodies were used: rabbit anti-PCH-2 (1:250) (Bhalla, 2008) , guinea pig anti-DSB-1 (1:500) (Rosu, 2013). All secondary antibodies were used at a 1:500 dilution and included: Alexa488 anti rabbit (Invitrogen) and Cy3 anti guinea pig (Jackson Immunochemicals).

Images of immunostaining experiments were obtained using a DeltaVision Personal DV system (Applied Precision) equipped with a 100x N.A. or 150x 1.40 oil-immersion objective (Olympus), resulting in an effective XY pixel spacing of 0.064 or 0.042 microns/pixel. Z-stacks were collected at 0.2-μm Z-spacing and processed by constrained, iterative deconvolution. Imaging, and image scaling were performed using functions in the softWoRx software package. Projections were generated using a maximum intensity algorithm. Composite images from immunostaining experiments were processed and quantified using Fiji.

**Figures and Statistics:**

Figures were assembled using Adobe Illustrator. All histograms and statistics were performed using Prism Graphpad. Tukey’s multiple comparison test was used to quantify significance.
